# Piperine attenuates fibrogenesis and inflammation in hepatic fibrosis by switching STAT3 phosphorylation at Ser727

**DOI:** 10.1016/j.chmed.2026.05.007

**Published:** 2026-05-10

**Authors:** Xu Dai, Yunuo Wu, Lihua Lian, Zhenyu Cui, Jixing Nan, Yanling Wu

**Affiliations:** Key Laboratory of Natural Medicines of the Changbai Mountain (Ministry of Education), Key Laboratory for Traditional Chinese Korean Medicine Research (State Ethnic Affairs), College of Pharmacy, Yanbian University, Yanji 133002, China

**Keywords:** extracellular matrix, hepatic fibrosis, piperine, *Piper nigrum* L., STAT3

## Abstract

**Objective:**

*Piper nigrum* is a medicinal and edible spice that is popular worldwide due to its unique spicy flavor, and is considered as a folk medicine for treating digestive system diseases. Piperine (PIP) is a bioactive alkaloid derived from *P. nigrum*, with remarkable hepatoprotective efficacy. Hepatic fibrosis represents a critical phase in the advancement of distinct chronic liver conditions towards cirrhosis, and currently lacks effective therapeutic drugs. The study aims to investigate the hepatoprotective functions of PIP and the underlying mechanism of PIP in combating hepatic fibrosis.

**Methods:**

C57BL/6J mice with hepatic fibrosis were induced by thioacetamide (TAA) and subsequently subjected to treatment with PIP or curcumin. Immortalized rat hepatic stellate cells (HSCs) were stimulated with transforming growth factor-*β* (TGF-*β*), followed by culture with PIP or niclosamide [signal transducer and activator of transcription 3 (STAT3) inhibitor], respectively. Human hepatic stellate cell line LX-2 were activated by TGF-*β* and transfected with specific small interfering RNA (siRNA) to silence *STAT3* gene, and afterwards cultured with PIP. The mouse AML-12 cells and macrophage-like murine cells Raw 264.7 were stimulated with lipopolysaccharide (LPS), followed by culture with PIP.

**Results:**

*In vivo*, PIP reduced serum transaminase levels, collagen deposition, and decreased the excessive accumulation of extracellular matrix (ECM), comprising *α*-smooth muscle actin (*α*-SMA), collagen type I (Collagen I), and tissue inhibitor of metalloproteinases-1 (TIMP-1)/matrix metalloproteinase 13 (MMP13) ratio expressions. PIP inhibited inflammatory cytokines release and myeloperoxidase (MPO) expression, including interleukin-1 receptor type 1 (IL-1R1), cysteine-aspartic acid protease-1 (Caspase-1), and IL-6. PIP specifically inhibited STAT3 phosphorylation at Ser727, downregulated phosphorylated janus kinase 2 (p-JAK2), and upregulated suppressor of cytokine signaling 3 (SOCS3) and protein inhibitor of activated STAT 1/3 (PIAS1/3) expressions. *In vitro*, PIP inhibited ECM deposition and inflammatory cytokines release in activated HSCs. PIP selectively suppressed p-STAT3 at Ser727 without affecting Tyr705, and modulated p-JAK2, SOCS3, and PIAS1/3 expressions in activated HSCs, function as a STAT3 inhibitor. PIP inhibited LPS‑induced M1 polarization of RAW 264.7 macrophages. PIP decreased Cleaved‑Caspase 3 expression and apoptotic cells in LPS‑induced AML‑12 hepatocytes. STAT3 deficiency amplified regulation of PIP on *α*-SMA and p-JAK2, as well as its upregulation on SOCS3 and PIAS1/3 in activated LX-2 cells.

**Conclusion:**

PIP improved hepatic fibrosis via inhibiting ECM excessive deposition and inflammatory secretion. Switching STAT3 phosphorylation at Ser727 might be the underlying targets for PIP against hepatic fibrosis, which provide an effective candidate and therapeutical strategy for hepatic fibrosis.

## 1 Introduction

Hepatic fibrosis is the reversible pathological event characterized by excessive accumulation of extracellular matrix (ECM), and ultimately progresses to hepatic cirrhosis and even hepatocellular carcinoma (Kisseleva and Brenner, 2021, Li et al., 2024). At present, there is no specific and effective treatment for liver fibrosis in clinic. The main approach is to alleviate liver injury and inflammation by treating underlying diseases that cause liver damage, and further improve liver fibrosis (Zhao, Tang, & Cheng, 2024). The most important treatment for liver fibrosis currently is etiological treatment. Although effective etiological treatment can slow down or even reverse liver fibrosis, etiological treatment cannot completely treat liver fibrosis or improve the prognosis of liver fibrosis patients (Mohammed, Attia, Mohamed, Elbaset, & Fayed, 2023). There are many targets and experimental drugs being researched and developed for the treatment of liver fibrosis, which are still in the preclinical and clinical trial stages (Manka, Zeller, & Syn, 2019). The development of anti-liver fibrosis drugs still faces great challenges, mainly due to the complex fibrogenesis process and mechanism, numerous influencing factors, and significant individual differences (Singh, Akhtar, & Shukla, 2025). Therefore, it’s still urgent to explore multi-target and multi mechanism anti-fibrotic drugs to more efficiently curb disease progression.

The immortalized rat hepatic stellate cells (HSCs) activation is the central driver in the pathogenesis of liver fibrosis, potentially leading to their transformation into myofibroblast-like cells, which contribute to the accumulation of ECM (Barry et al., 2020, Dou et al., 2024). Focusing on the activation of HSC represents a hopeful treatment approach for reversing hepatic fibrosis, given its dynamic and reversible characteristics (Kamm and McCommis, 2022, Saluja et al., 2024).

The activation of the janus kinase 2 (JAK2)/signal transducer and activator of transcription 3 (STAT3) signaling pathway is a crucial indicator of HSCs activation and involved in the progression of liver fibrosis (Xiang et al., 2018). The inhibition of JAK2/STAT3 signaling pathway impedes the trans-differentiation of HSC, and diminishes the expressions of genes associated with fibrosis (Xin et al., 2020, Zhou et al., 2022). Emerging studies demonstrate that the phosphorylation of STAT3 at Ser727 and Tyr705 collectively accelerates the onset and progression of cancer (Yang et al., 2020, He et al., 2022). However, the role of STAT3 phosphorylation at Tyr705 or Ser727 in the progression of hepatic fibrosis remains in doubt. Thus, targeting STAT3 and its cascade reaction could offer valuable insights into therapeutic strategies for hepatic fibrosis treatment.

**Fig. 1:**
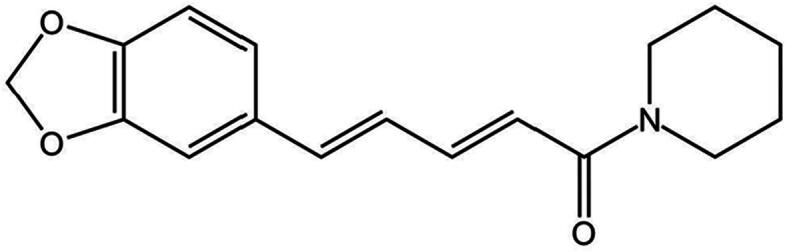
Chemical structure of PIP.

In recent years, the scientific community has increasingly focused on traditional Chinese medicine (TCM) due to its distinctive therapeutic advantages, including remarkable efficacy, low toxicity profile, and minimal adverse effects (Wei et al., 2022, Jin et al., 2025). Revered as the “king of spices” and “black gold”, *Piper nigrum* L. ranks among the most commonly utilized spices and a folk medicine to treat digestive system diseases (Lu et al., 2023). “*Compendium of Materia Medica*” recorded that *P. nigrum* could coordinate digestive system and harmonize the five zang-organs. Piperine (PIP, the chemical structure in Fig. 1) is the amide alkaloid compound mainly isolated from *P. nigrum* and has analgesic, antipyretic, anti-diarrheal and anticonvulsant effects (Yadav et al., 2023). Growing evidence indicates that PIP could act as a promising therapeutic agent for liver diseases (Azam, Park, Kim, & Choi, 2022). A clinic trial showed that the combined administration of curcumin and PIP notably ameliorated hepatic function and lipid profiles in individuals diagnosed with non-alcoholic fatty liver disease (NAFLD) (Panahi et al., 2019). Nevertheless, the function of PIP in hepatic fibrosis remains unclear. The studies mentioned suggest that PIP may offer hepatoprotective benefits regarding hepatic fibrosis, and it is worth exploring its therapeutic effects and potential mechanisms for liver fibrosis.

The study investigated whether PIP would inhibit HSCs activation and attenuate fibrogenesis and inflammation in hepatic fibrosis mice by switching STAT3 phosphorylation at Ser727 and following cascade reaction, and providing a theoretical foundation for therapeutic strategies and candidate for liver fibrosis in the future.

## 2 Material and methods

### 2.1 Materials and reagents

PIP (purity > 98%, high performance liquid chromatography grade, lot number: E1824063) and curcumin (purity > 98%, high performance liquid chromatography grade, lot number: L2522084) were supplied by Chengdu Derick Biotechnology Co., Ltd. (Chengdu, China). Niclosamide (STAT3 inhibitor, lot number: M30GB150274) was obtained from Yuanye biological (Shanghai, China). Thioacetamide (TAA, lot number: C1925146) was sourced from Sigma-Aldrich (St. Louis, USA), while transforming growth factor-*β* (TGF-*β*, lot number: 1217354) was obtained from PeproTech (Cranbury, USA). Bioss (Woburn, USA) supplied primary antibodies against collagen type I (Collagen I, bs-0578R, lot number: AG05161874), *α*-smooth muscle actin (*α*-SMA, bs-10196R, lot number: BD11292299), fluorescein isothiocyanate (FITC, bs-0296G-FITC, lot number: BD01311772) and phycoerythrin (PE, bs-0296G-PE-Cy5, lot number: BD11151342). Abcam (Cambridge, USA) provided primary antibodies against tissue inhibitor of metalloproteinases 1 (TIMP-1, ab-61224, lot number: F1119), matrix metalloproteinase 13 (MMP13, ab-75606, lot number: D0721), STAT3 (ab-83805, lot number: G1411), phosphorylated-STAT3 (p-STAT3, Y705, ab-76315, lot number: F1544), p-STAT3 (Ser727, ab-86430, lot number: F4904), suppressor of cytokine signaling 3 (SOCS3, ab-16030, lot number: F1825), JAK2 (ab-108596, lot number: K1128), phosphorylated-JAK2 (p-JAK2, Y1007 + Y1008, ab-32101, lot number: K1059), glyceraldehyde-3-phosphate dehydrogenase (GAPDH, ab-8245, lot number: F1211) and 4′,6-diamidino-2-phenylindole (DAPI, lot number: 113782-12). Primary antibodies against myeloperoxidase (MPO, sc-390109, lot number: E0620), cysteine-aspartic acid protease 1 (Caspase-1, sc-56036, lot number: R3077), interleukin-1 receptor type 1 (IL-1R1, sc-393998, lot number: G3839), IL-6 (sc-57315, lot number: G2822), protein inhibitor of activated STAT1/3 (PIAS1/3, sc-271172, lot number: J2921), control-small interfering RNA (siRNA, sc-37007, lot number: D0325), *STAT3*-siRNA (sc-29493, lot number: E0217), anti-inducible nitric oxide synthase (iNOS, sc-7271, lot number: E1124) and anti-cluster of differentiation 206 (CD206, sc-34577, lot number: E0123) were acquired from Santa Cruz Biotechnology Inc. (Dallas, USA). Additionally, the primary antibody against IL-1*β* (AF-401-NA, lot number: NP3521052) was procured from R&D Systems (Minneapolis, USA). The fetal bovine serum (FBS, lot number: 1869024), Dulbecco’s Modified Eagle Medium (DMEM, lot number: 3256031), DMEM/F12 medium (lot number: 3207739) and opti-MEM®I (1 ×, lot number: 3149774) were obtained from Gibco (Waltham, USA). The streptomycin sulfate (lot number: 325E0525), penicillin G sodium salt (lot number: C11955477), and 3-(4,5-dimethylthiazol-2-yl)-2,5-diphenyl-tetrazolium bromide (MTT, lot number: 54240622008) were obtained from Solarbio (Beijing, China). Thermo Fisher Scientific (Waltham, USA) provided Lipofectamine® RNAiMAX (lot number: 2218549). The bicinchoninic acid (BCA) protein assay kit (lot number: P0009) and erminal deoxynucleotidyl transferase-mediated dUTP-biotin nick end labeling (TUNEL) kit (lot number: 092120240209) were obtained from Beyotime (Shanghai, China). Horseradish peroxidase (HRP)-conjugated goat anti-mouse IgG (H + L) antibody (K1221, lot number: 25002 K1211) and goat anti-rabbit IgG (H + L) antibody (K1223, lot number: 25001K1223) were purchased from APExBIO (Houston, USA). Eastep® super total RNA extraction kit (lot number: LS1040) was obtained from Promega (Madison, USA). Abways (Shanghai, China) provided the primary antibody against p-STAT3 (Ser727, CY5291, lot number: F153403) and B-cell lymphoma-extra large (Bcl-xL, CY5050, lot number: F091207). Roscovitine (Rosco, lot number: G2220199, 5 mg) was obtained from Moligand^TM^ (Shanghai, China). Alanine aminotransferase (ALT, lot number: C009-2-1) and aspartate aminotransferase (AST, lot number: C010-2-1) were obtained from Changchun HuiLi Biological Co., Ltd. (Changchun, China). Solarbio (Beijing, China) provided hematoxylin and eosin (HE, lot number: G1080) and Sirius Red (lot number: 2306001). 10% Hydrogen peroxide (lot number: ZLI-9311) was procured from ZSGB-BIO Biotechnology Co., Ltd. (Beijing, China). Goat serum (lot number: abs933) was obtained from Absin (Shanghai, China). MaxVision^TM^ HRP-Polymer IHC kit (lot number: KIT-5920) was obtained from Maixin Biol (Fuzhou, China). InvivoGen (Toulouse, France) provided lipopolysaccharide (LPS, lot number: LEB-41-02). 4% Paraformaldehyde (lot number: E672002-0500) was purchased from Sangon Biotech (Shanghai, China). 0.1% Triton X-100 (lot number: 2527642B) was obtained from Thermo Fisher Scientific Inc. (Waltham, USA). Phosphate buffered saline (PBS, lot number: L205-500) was purchased from BioChannel Biotechnology Co., Ltd. (Hangzhou, China). Absin (Shanghai, China) provided RIPA lysis buffer (lot number: 2500120013). SDS-PAGE (lot number: WB2102) was purchased from Biotides Biotechnology Co., Ltd. (Beijing, China). Chemiluminescence (ECL, lot number: 180-5001) was obtained from Tanon (Shanghai, China). 3,3′-diaminobenzidine (DAB, lot number: DW046DBTL1232) chromogen was obtained from Elabscience (Wuhan, China).

### 2.2 Animal procedures and treatments

Specific pathogen free (SPF) male C57BL/6J mice with a body age from 6 to 8 weeks and body weight of 20–22 g were purchased from Changchun YISI Experimental Animal Institute for Biological Sciences (SCXK [JI] 2020–0002, Changchun, China). Mice were kept in controlled environments with a temperature range of 21–22 °C and a light/dark cycle of 12 h, while having unrestricted access to tap water and food. All procedures related to the care and experimentation of animals were carried out in accordance with the protocols established by the National Institutes of Health (NIH) in their publication, “Guide for the Care and Use of Laboratory Animals”. Approval for study was granted by the Animal Ethics Committee at Yanbian University (approval number: YD20250122011).

After acclimatization for one week, the mice were randomly divided into the following seven groups (*n* = 6): the normal group, TAA group, TAA + PIP (15, 30 and 60 mg/kg) groups, TAA + curcumin (Cur, 20 mg/kg) groups, and PIP single group (60 mg/kg), respectively. Mice with hepatic fibrosis were induced with intraperitoneal injection of TAA at 100 mg/kg, three times in the first week, and 200 mg/kg in the next four weeks, two times per week. Notably, the animal model of hepatic fibrosis induced by TAA has good repeatability and is similar to human hepatic fibrosis, and its dose is based on previous related studies (Song et al., 2020). The normal group mice were injected with equal volume of sterile saline. Except for the normal and TAA group, mice in PIP or Cur groups were administered by gavage with PIP or Cur for five weeks, respectively. Curcumin is often used as a positive control because it slows down the pathological progression of hepatic fibrosis and is linked to less collagen buildup (Rivera-Espinoza & Muriel, 2009). The doses of PIP (15, 30, and 60 mg/kg) were chosen according to the regimen reported in previous studies demonstrating its hepatoprotective effects (Coelho et al., 2024). At the conclusion of the experiments, anesthesia was administered using 3% sodium pentobarbital to sacrifice the mice, then the liver tissues and blood were collected by cardiac puncture for subsequent experiments. The liver tissues were preserved in 10% neutral buffered formalin for histological examination, while the remaining samples were kept in −80 °C freezers until additional analysis.

### 2.3 Serum biochemical assays

Centrifuge the collected serum samples at 4 °C and 1 000 r/min for 30 min. After centrifugation, collect the supernatant and measure the ALT and AST levels by using kits, following the instructions provided by the manufacturer. The results were visualized with the help of an Autodry Chemistry Analyzer (SPOTCHEM^TM^ SP4410, Arkray, Kyoto, Japan).

### 2.4 Histopathological examination

Liver tissues were preserved in 10% neutral buffered formalin, dehydrated using a sequential ethanol series, and embedded in paraffin. The tissues were cut into 5 μm sections, and stained with HE and Sirius Red in order to evaluate architecture alterations and status of the fibrous areas. For immunohistochemical analysis, sections were performed antigen repair, endogenous peroxidase blocking up with 10% hydrogen peroxide and non-specific site blocking up with 5% goat serum. The primary antibodies at a dilution of 1:200 were incubated overnight at 4 °C, which was then followed by incubation with secondary antibodies conjugated to HRP. The presence of immunoreactivity was demonstrated by employing DAB chromogen, following the instructions provided by the manufacturer. Finally, the sections were counterstained with hematoxylin and subsequently mounted using neutral gum. The stained sections were observed and photographed under a light microscope (Olympus, Tokyo, Japan).

### 2.5 Cell culture and treatment

The rat HSC-T6 hepatic stellate cell line was kindly provided by Prof. Jung Joon Lee (Korea Research Institute of Biotechnology, Daejeon, Korea) and the human LX-2 hepatic stellate cell line was purchased from American Type Culture Collection (ATCC, Manassas, USA). The mouse AML-12 cell line and macrophage-like murine cell line Raw 264.7 were obtained from Fuheng Biology (Shanghai, China). The LX-2 and HSC-T6 cells were cultured in DMEM, supplemented with 10% FBS, along with 1% streptomycin and penicillin, maintaining an environment of 37 °C and 5% CO_2_. The rat HSC-T6 cells were activated by TGF-*β* stimulation (10 ng/mL) for 2 h and subsequently treated with PIP at concentrations of 3.125, 6.25 and 12.5 μmol/L, or niclosamide at 40 μmol/L as previously described (Cui et al., 2021), or roscovitine at 5 μmol/L for 6 h. Previous studies confirmed that roscovitine administration protects against liver diseases through inhibition of macrophage inflammatory actions and HSC activation at the onset of liver injury (Liu et al., 2021).

AML-12 cells were incubated in DMEM/F12 medium and Raw 264.7 cells were cultured in DMEM supplemented with penicillin (100 U/mL), streptomycin (100  mg/mL), 10% FBS at 37 °C, under 5% CO_2_. AML-12 cells and Raw 264.7 cells were cultured in 6-well plates at a density of 1 × 10^6^ per well, and grown to full adherence for 24 h. AML-12 cells were incubated with LPS (100 ng/mL) for 6 h and subsequently treated with PIP at concentrations of 12.5 μmol/L for 6 h. Raw 264.7 were incubated with LPS (100 ng/mL) for 2 h and subsequently treated with PIP at concentrations of 12.5 μmol/L for 6 h. The concentration of PIP was set according to the experimental results of MTT.

### 2.6 Cell viability assay by MTT

The HSC-T6 cells was seeded at a density of 1 × 10^4^ per well in a 96-well plate and treated with PIP (0 − 200 μmol/L) for 24 h. Afterward, a solution of MTT was introduced into the cells and incubated for 3 h, followed by recording the absorbance at 570 nm through a microplate reader. The viability rate of cells was calculated according to this formula: Cell viability (%) = (*A*_treated_ − *A*_blank_)/(*A*_normal_ − *A*_blank_) × 100.

### 2.7 Transfection of siRNA in vitro

The LX-2 cells were transfected with siRNA using Lipofectamine® RNAiMAX reagent. Specifically, control siRNA (sicontrol) at 2 μmol/L and *STAT3* siRNA (si*STAT3*) at 10 μmol/L were prepared in RNA-free water according to the manufacturer’s protocol. Silencing reagents were prepared in super-clean worktable: 500 μL DMEM + 15 μL sicontrol or 15 μL si*STAT3* + 5 μL Lipofectamine® RNAiMAX in a 1.5 mL centrifuge tube. The mixture was gently shaken and incubated at room temperature for 15 min. During the incubation of the silencing reagent, LX-2 cells were seeded at a density of 1 × 10^4^ cells/well in a 6-well plate, followed by the addition of the pre-incubated silencing reagent for 48 h. Before transfection finishing, LX-2 cells were stimulated with TGF-*β* (10 ng/mL) for 2 h, followed by incubation with or without PIP (12.5 μmol/L) for 6 h. Then, total cellular proteins were then extracted for subsequent analyses.

### 2.8 Immunofluorescence staining

The liver tissue sections (5 μm thickness) were preserved in an acetone-methanol solution (1:1, *v*/*v*). Meanwhile, the cells seeded in glass bottom cell culture dishes were fixed by 4% paraformaldehyde. Following fixation, samples were permeabilized with 0.1% Triton X-100 in PBS for 10 min on ice cubes, then blocked with 5% goat serum for 1 h at room temperature. Primary antibodies were applied and incubated overnight at 4 °C, then followed by 1 h incubation with species-specific secondary antibodies at room temperature. Nuclei were stained with DAPI following the protocol provided by the manufacturer. Fluorescence images were captured utilizing a fluorescence microscope (FV10i, Olympus, Tokyo, Japan).

### 2.9 Western blotting analysis

After the above processing, extract HSCs-T6 cells and liver tissues lysate samples using a mixture of protease inhibitor and RIPA lysis buffer. A BCA protein assay kit was then used to estimate the cell lysate protein concentrations. Equal quantities of proteins were separated via 8%–12% SDS-PAGE electrophoresis and thereafter, transferred to PVDF membranes. The membranes were incubated with blocking buffer (5% skim milk) at room temperature for 1 h, and then incubated with the primary antibody at a dilution of 1:1 000 overnight at 4 °C, followed by incubation with the corresponding secondary antibody at a dilution of 1:8 000 at room temperature for 2 h. Finally, the immunoreactive bands were evaluated using ECL and recorded on X-ray film. Western blotting grayscale analysis was performed using Image-J software for quantification, and the relative expression level was obtained by calculating the integrated optical density (IOD) ratio of the target protein to the reference protein. Relative protein expression levels were normalized to that of GAPDH.

### 2.10 Real-time PCR (RT-PCR) analysis

Total RNA extraction from liver tissue or cells was performed using the Eastep® Super Total RNA Extraction kit, in accordance with the protocol provided by manufacturer. cDNA was synthesized using reverse transcriptase, and then conventional PCR amplification was performed with specific primers. According to the recipe in the instruction manual of RT-PCR, the products were run on a 2% agarose gel, stained with ethidium bromide. Band intensities were quantified using Image‑J software. Relative expression levels of target genes were normalized to the internal reference gene *Gapdh* (mouse) or *GAPDH* (human) and calculated as the ratio of the target gene band intensity to that of the reference gene. For reverse transcription PCR, the specified primers shown in Table 1, Table 2 were utilized.

**Table 1:** Sequences of mouse primers used in RT-PCR.

Genes	Primer sequences (5′-3′)	
	Forward	Reverse
*Gapdh*	CTTGTGCAGTGCCAGCC	GCCCAATACGGCCAAATCC
*Acta2*	CATCAGGGAGTAATGGTTGG	CACAATACCAGTTGTACGTC
*Col1a1*	TGAGTCAGCAGATTGAGAAC	TACTCGAACGGGAATCCA
*Timp1*	GGAAAGCCTCTGTGGATATG	AACAGGGAAACACTGTGC
*Tnf*	TCACACTCAGATCATCTTCTC	AGACTCCTCCCAGGTATATG
*Il1b*	CACAGGCTCTCTTTGAACAG	ACATCCTTCATCCTCAGAAAC
*Il6*	TCCTCTCTGCAAGAGACTTC	CCAGTTTGGTAGCATCCATC
*Il1a*	CTTGAGTCGGCAAAGAAATC	GAGATGGTCAATGGCAGAAC

**Table 2:** Sequences of human primers used in RT-PCR.

Genes	Primer sequences (5′-3′)	
	Forward	Reverse
*GAPDH*	GGCTCTCCAGAACATCATC	CTCTTCCTCTTGTGCTCTTG
*ACTA2*	TATGCCTCTGGACGCACAAC	CACGCTCAGCAGTAGTAACG
*COL1A1*	CAAGACGAAGACATCCCAC	CGGTTGATTTCTCATCATAGC
*TIMP1*	AATTCCGACCTCGTCATCAG	CATTCCTCACAGCCAACAGT
*TNF*	GAGCACTGAAAGCATGATCC	GAGGGTTTGCTACAACATGG
*IL1B*	CCTGTACGATCACTGAACTG	TTGGGATCTACACTCTCCAG
*IL6*	CAGGAGAAGATTCCAAAGATG	AAGTCTCCTCATTGAATCCAG
*IL1A*	CAGTGAAATTTGACATGGGTG	CAGGCATCTCCTTCAGCAG
*Caspase-1*	ACATCCTTCATCCTCAGAAAC	GATAATGAGGGCAAGACGTG
*STAT3*	CTGCTGCTGATCCTGTGGAC	CGGACTGGATCTGGGTCTTC

### 2.11 Molecular docking simulation

Molecular docking was employed to investigate the binding mode of PIP to STAT3. The crystal structures of STAT3 were obtained from the Protein Data Bank (PDB, https://www.rcsb.org/pdb/). Additionally, the structures of PIP were acquired from the PubChem database (https://pubchem.ncbi.nlm.nih.gov). The target protein and ligand compound were prepared following standard procedures. Subsequently, these files were subjected to AutoDock Vina (version 1.2.0, The Scripps Research Institute, La Jolla, USA) for analysis.

### 2.12 TUNEL staining

To detect the apoptosis in AML-12 cells, the cells were detected by TUNEL assay according to the manufacturer’s instructions. Following fixation with 4% paraformaldehyde, the cells were permeabilized using 0.3% Triton X-100 and subsequently incubated TUNEL assay mixture at 37 °C for 1 h. DAPI was used to stain nuclei. TUNEL positive cells were visualized with a fluorescence microscope.

### 2.13 Flow cytometry assay

RAW264.7 cells were re-suspended and incubated for 30 min in a solution of 1 × PBS. Following three washes with PBS, the cells were incubated with a 1:300 dilution of either anti-iNOS or anti-CD206 for 60 min. Afterward, the cells underwent three additional washes with PBS and then resuspended in 90 μL of PBS containing 10 μL of FITC-conjugated secondary antibody or PE-conjugated secondary antibody, incubating for 30 min in the dark at room temperature. Flow cytometry (BD Accuri^TM^ C6 Plus, San Jose, USA) analysis was utilized to quantify the macrophages.

### 2.14 Statistical analysis

Statistical analysis was conducted using Prism (Version 8.4.3) and indicated as mean ± standard deviation (SD). Before ANOVA analysis, the data was subjected to Levene’s homogeneity of variance test, and it was found that the obtained data had uniform variance. For data that met homogeneity of variance, one-way ANOVA followed by Tukey’s multiple comparison test was used. Post hoc tests were only carried out in multi-group comparisons of parametric variables if there existed no significant variance in homogeneity and if *F* upon ANOVA reached. All data met the requirements of the statistical test. *P* < 0.05 was considered statistically significant between datasets.

## 3 Results

### 3.1 PIP ameliorated serum transaminases and collagen accumulation in mice with TAA-induced liver fibrosis

**Fig. 2:**
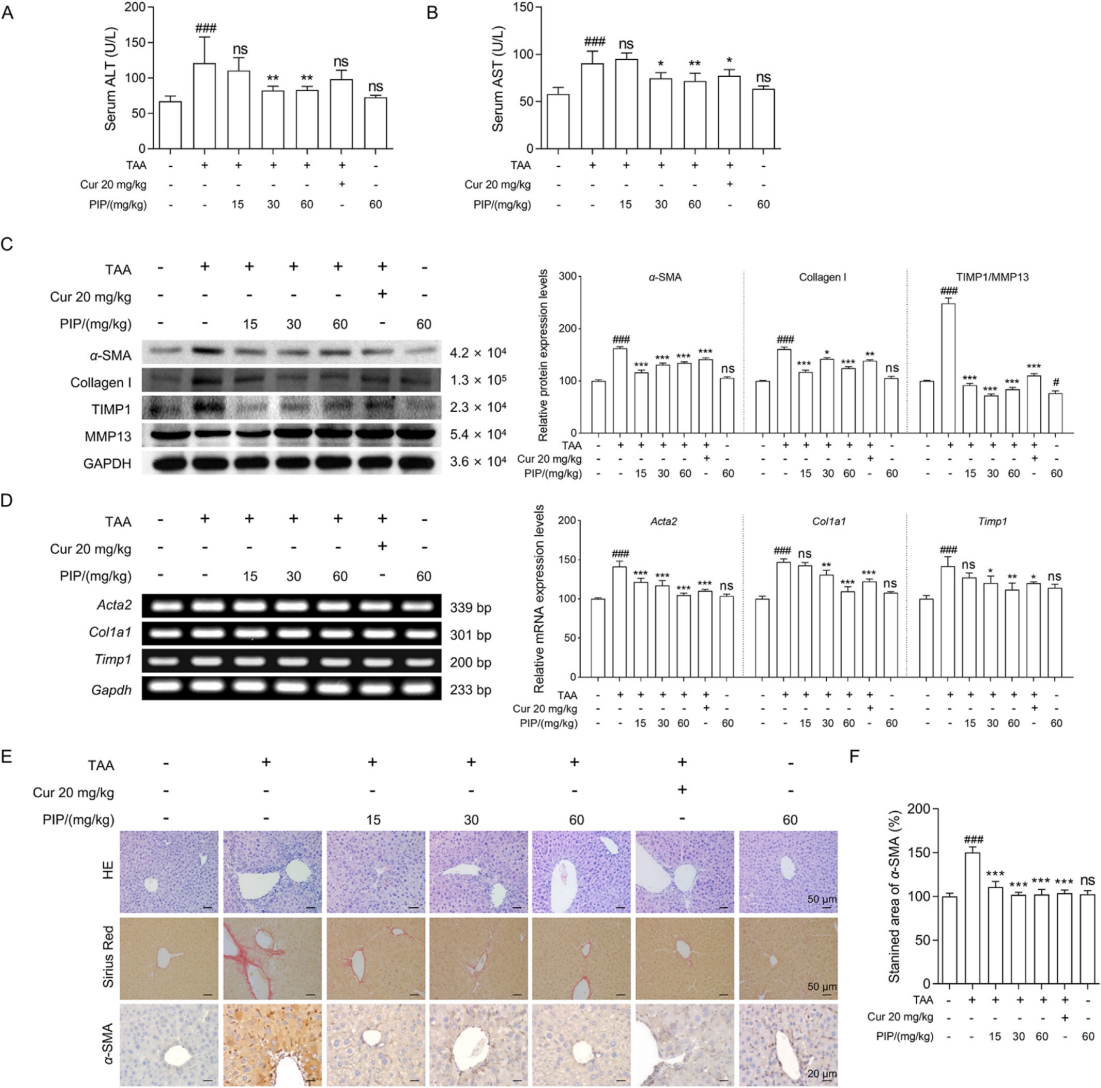
PIP ameliorates liver injury and hepatic fibrosis in TAA-induced mice. (A, B) Serum ALT and AST. (C) Protein expressions and quantitative results of *α*-SMA, Collagen I, TIMP-1/MMP13. (D) Representative RT-PCR analysis for expressions of *Acta2*, *Col1a1*, and *Timp1*. (E) Liver appearance pictures and representative HE and Sirius Red staining images (scale bar: 50 μm, ×200 magnification). Immunohistochemical staining for *α*-SMA (scale bar: 20 μm, ×400 magnification). (F) Integrated optical densities of *α*-SMA was quantified using Image-J. Each band from Western blotting analysis was normalized against GAPDH (*n* = 3). RT-PCR analysis was normalized against *Gapdh* (*n* = 3). ^#^*P* < 0.05, ^###^*P* < 0.001 *vs* normal group; *P < 0.05, ^**^P < 0.01, ^***^P < 0.001 *vs* TAA group; ns, no significance.

In the hepatic fibrosis mice, TAA injection notably raised the serum ALT and AST levels, while PIP (30 and 60 mg/kg) could obviously decrease serum transaminase (Fig. 2A and B). The protein expressions of *α*-SMA, Collagen I, TIMP-1 and TIMP-1/MMP13 ratio in TAA group were notably increased in comparison to the normal group, while PIP or Cur could reduce these expressions and PIP had a better inhibitory effect. Single PIP showed no significance on the protein expressions of *α*-SMA and Collagen I compared with normal group (Fig. 2C). PIP or Cur significantly decreased the mRNA expressions of *Acta2*, *Timp1*, and *Col1a1* caused by TAA. The effect of PIP (60 mg/kg) on reducing mRNA expression was more pronounced compared with Cur. PIP single group exhibited no notable difference compared with the normal group (Fig. 2D). HE staining and Sirius Red revealed that TAA caused hemorrhagic necrosis with infiltration of inflammatory cells and collagen accumulation in mice, while PIP or Cur obviously ameliorated these pathological alterations. In addition, immunohistochemical staining indicated that PIP or Cur significantly reduced the positive expression of *α*-SMA (in brown) induced by TAA (Fig. 2E and G). These results suggested PIP effectively mitigated collagen deposition and liver injury in mice induced by TAA.

### 3.2 PIP reduced neutrophil infiltration and regulated inflammation in mice induced by TAA

**Fig. 3:**
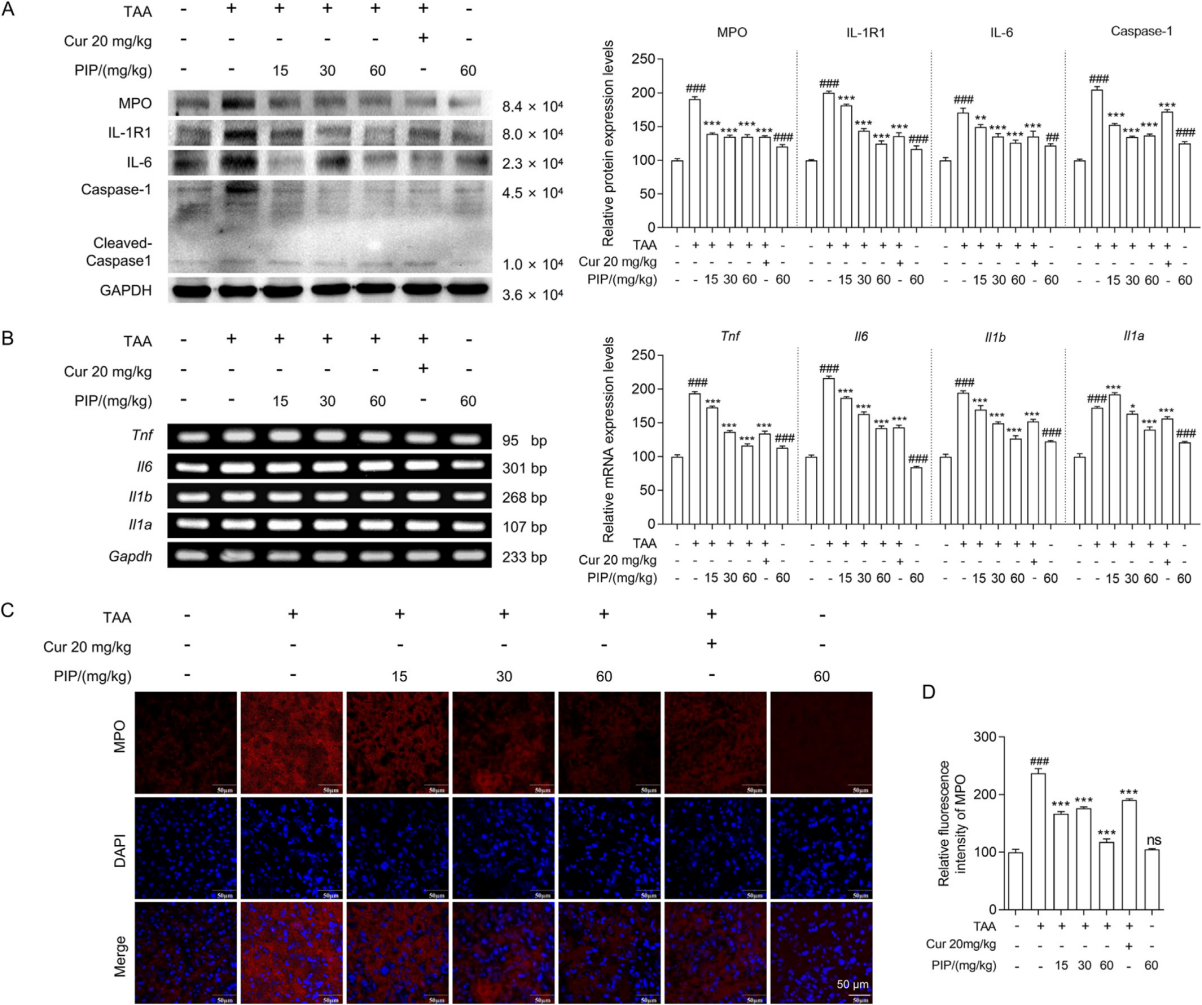
PIP attenuates the levels of proinflammatory cytokines induced by TAA in mice. (A) Protein expressions and quantitative results of MPO, IL-1R1, IL-6, Caspase-1, cleaved-Caspase1. (B) Effect of PIP on mRNA expressions of *Tnf*, *Il6*, *Il1b* and *Il1a* in TAA-induced mice. (C) Immunofluorescence staining for MPO (red, ×600, scale bar: 50 μm), DAPI (blue, ×600, scale bar: 50 μm) and merge presents (×600, scale bar: 50 μm). (D) Relative fluorescence intensity of MPO was quantified using Image-J. Each band from Western blotting analysis was normalized against GAPDH (*n* = 3). RT-PCR analysis was normalized against *Gapdh* (*n* = 3). ^###^*P* < 0.001 *vs* normal group; *P < 0.05, ^***^P < 0.001 *vs* TAA group; ns, no significance.

MPO is regarded as an indicator of neutrophil function and activation and released into the inflammatory site with neutrophil infiltration (Rizo-Téllez, Sekheri, & Filep, 2022). TAA significantly increased MPO, IL-1R1, IL-6 and Caspase-1 protein expressions in comparison to the normal group. PIP or Cur notably reduced the upregulation of MPO, IL-1R1, IL-6 and Caspase-1 protein expressions caused by TAA. In addition, PIP had a stronger ability to reduce the expression of these proteins (Fig. 3A). In comparison to the normal group, the mRNA expressions of *Tnf*, *Il6*, *Il1b* and *Il1a* were obviously upregulated in the TAA group, while PIP or cur notably downregulated the mRNA expression levels of *Tnf*, *Il6*, *Il1b* and *Il1a*. Moreover, the reduction effect of PIP (60 mg/kg) was better than Cur (Fig. 3B). Immunofluorescence staining further validated that PIP or Cur could diminish the elevation of MPO (in red) positive expression triggered by TAA. Single PIP showed no significance on MPO positive expression compared with normal group (Fig. 3C and D). The above results suggested that PIP could effectively inhibit hepatic inflammation induced by TAA.

### 3.3 PIP attenuated hepatic fibrosis via JAK2/STAT3 signaling pathway in mice induced by TAA

**Fig. 4:**
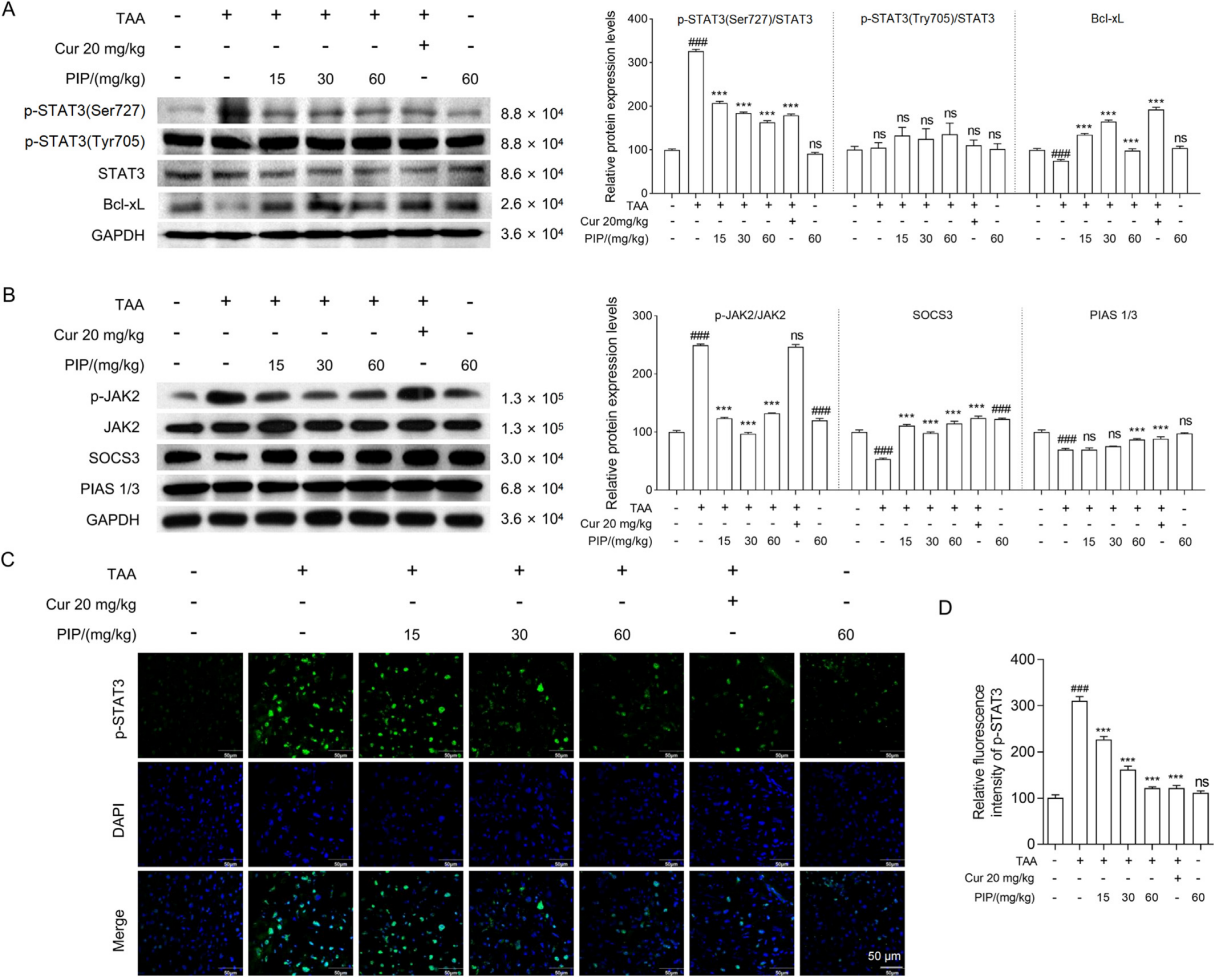
PIP alleviates TAA-induced liver fibrosis in mice via the JAK2/STAT3 signaling pathway. (A) Protein expressions and quantitative results of p-STAT3 (Ser727)/STAT3, p-STAT3 (Tyr705)/STAT3 and Bcl-xL. (B) Protein expressions and quantitative results of p-JAK2/JAK2, SOCS3 and PIAS1/3. (C) Immunofluorescence staining for p-STAT3 (green, ×600, scale bar: 50 μm) and DAPI (blue, ×600, scale bar: 50 μm) and merge presents (×600, scale bar: 50 μm). (D) Relative fluorescence intensity of p-STAT3 was quantified using Image-J. Each band from Western blotting analysis was normalized against GAPDH (*n* = 3). ^###^*P* < 0.001 *vs* normal group; ^***^P < 0.001 *vs* TAA group; ns, no significance.

The JAK2/STAT3 signaling pathway is crucial in combating liver fibrosis. TAA markedly elevated the protein expression of p-STAT3 (Ser727) in comparison to the normal group, while PIP or Cur obviously diminished the expression of p-STAT3 (Ser727). PIP single group exhibited no notable difference compared with the normal group. The protein expressions of total STAT3 and p-STAT3 (Tyr705) showed no significant change underlying TAA, PIP or Cur. Bcl-xL, a key downstream target of the JAK2/STAT3 pathway, is directly regulated by activated STAT3 and essential for promoting cell survival (Khan et al., 2024, Dai et al., 2025). TAA significantly decreased the protein expression of Bcl-xL compared with normal group. PIP or Cur notably increased the protein expression of Bcl-xL compared with TAA group (Fig. 4A). Single PIP showed no significance compared with normal group. In addition, p-JAK2 protein expression was notably upregulated in the TAA group in comparison to the normal group. PIP obviously down-regulated the protein expression of p-JAK2. SOCS3 is considered a feedback regulator for STAT3 and PIAS proteins play an essential role in JAK/STAT. TAA decreased SOCS3 and PIAS1/3 protein expressions in comparison to the normal group, while PIP (60 mg/kg) or Cur significantly improved the protein expressions of SOCS3 and PIAS1/3 (Fig. 4B). Immunofluorescence staining revealed that the positive expressions of p-STAT3 (in green) were increased in mice induced by TAA compared with normal group, and these expressions were reversed by PIP or Cur (Fig. 4C and D). The results indicated that PIP has the potential to reduced liver fibrosis triggered by TAA through the modulation of the JAK2/STAT3 signaling pathway.

### 3.4 PIP reduced ECM deposition and inhibited TGF-β-stimulated HSCs cells activation

**Fig. 5:**
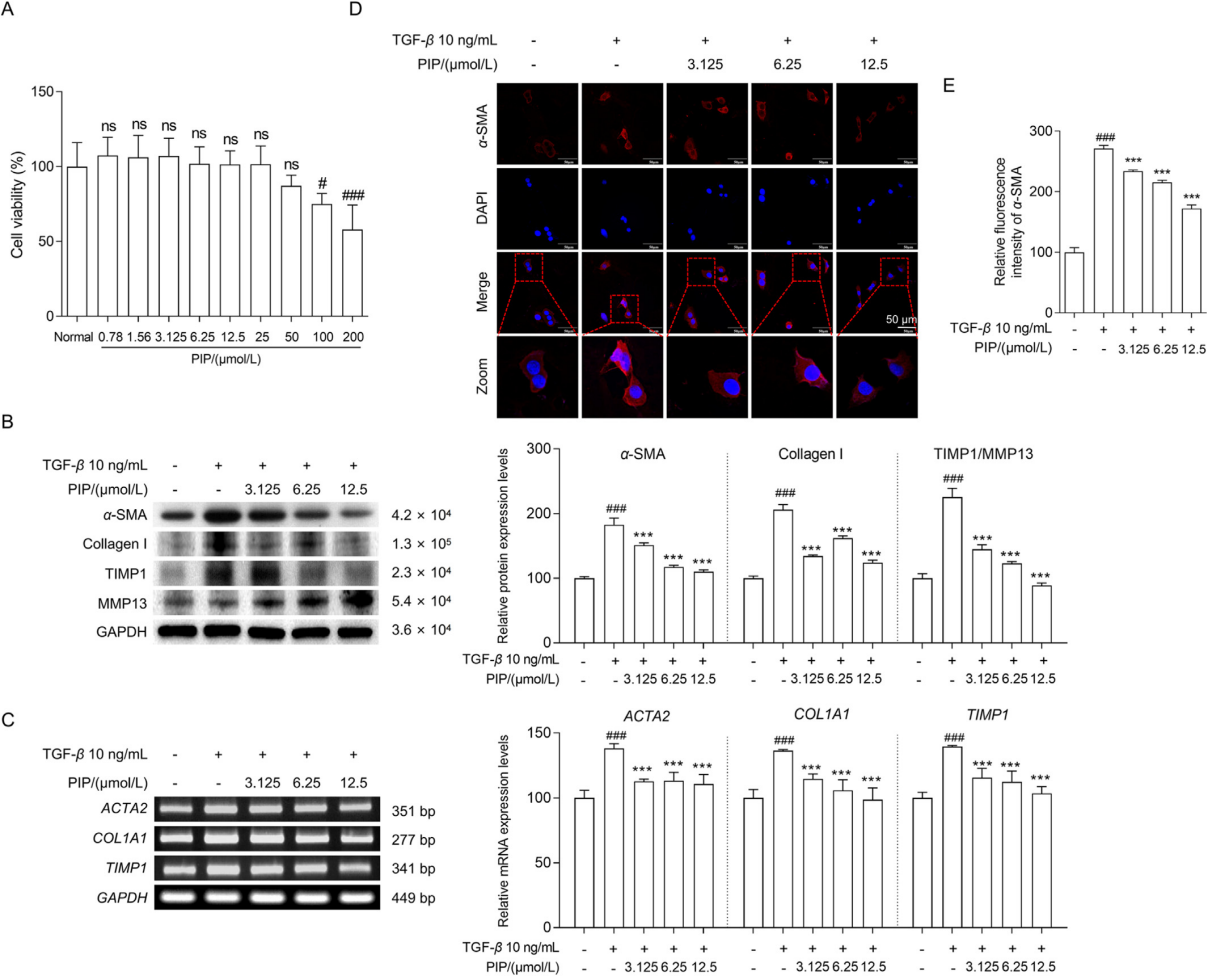
PIP inhibited ECM accumulation in activated HSC-T6 cells. (A) Cell viability of HSC-T6 cells with PIP by MTT assay. (B) Protein expressions and quantitative results of *α*-SMA, Collagen I, TIMP-1/MMP13 in HSC-T6 cells. (C) Representative RT-PCR analysis for expressions of *ACTA2*, *COL1A1*, and *TIMP1* in LX-2 cells. (D) Immunofluorescence staining for *α*-SMA (red, ×600, scale bar: 50 μm), DAPI (blue, ×600, scale bar: 50 μm), merge presents (×600, scale bar: 50 μm) and zoom presents in HSC-T6 cells. (E) Relative fluorescence intensity of *α*-SMA was quantified using Image-J. Each band from Western blotting analysis was normalized against GAPDH (*n* = 3). RT-PCR analysis was normalized against *GAPDH* (*n* = 3). ^###^*P* < 0.001 *vs* normal group; ^***^P < 0.001 *vs* TGF-*β* group; ns, no significance.

MTT assay showed that PIP did not notably influence the viability of HSC-T6 cells at concentrations of 0.78–50 μmol/L, and obviously decreased cell viabilities at concentrations of 100 and 200 μmol/L (Fig. 5A). Therefore, the concentrations of PIP (3.125, 6.25, and 12.5 μmol/L) were used in the subsequent experiments. In TGF-*β*-stimulated HSC-T6 cells, the protein expressions of *α*-SMA, Collagen I and TIMP-1/MMP13 ratio were notably increased in comparison to the normal group, while PIP effectively decreased these protein expressions (Fig. 5B). Similarly, RT-PCR showed that TGF-*β* could upregulate the mRNA expressions of *ACTA2*, *COL1A1*, and *TIMP1* in LX-2 cells, while PIP obviously downregulated the mRNA expressions of *ACTA2*, *COL1A1*, and *TIMP1* (Fig. 5C). Immunofluorescence staining demonstrated that PIP prominently diminished the positive expression of *α*-SMA (in red) in HSC-T6 cells stimulated by TGF-*β* (Fig. 5D and E). The results demonstrated that PIP might has the ability to modulate the balance of ECM and suppress the activation of HSCs.

### 3.5 PIP reduced release of inflammatory cytokines in TGF-β-stimulated HSCs and apoptosis in LPS-induced AML-12 cells, and inhibited macrophage polarization

**Fig. 6:**
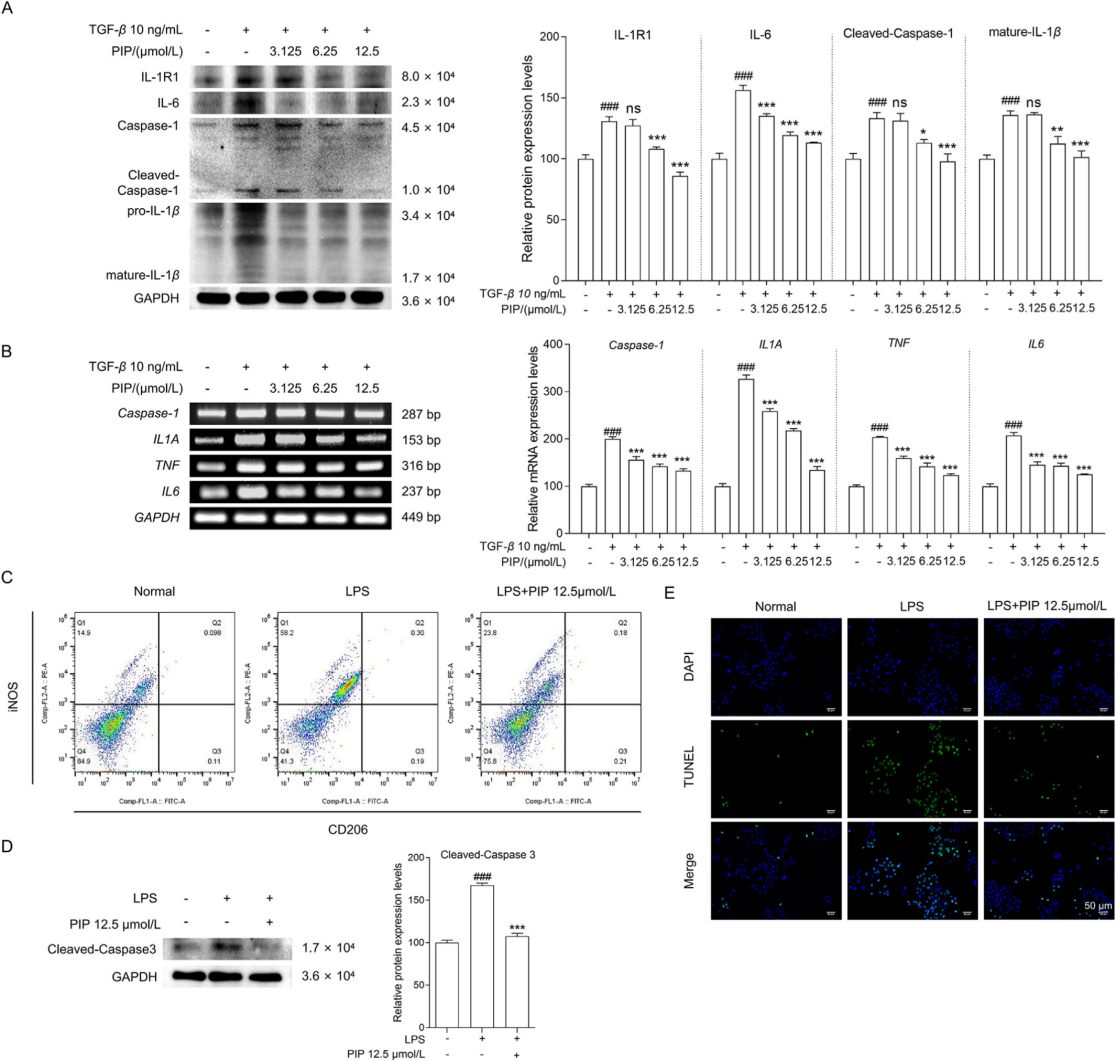
PIP reduced the release of inflammatory cytokines in TGF-*β*-stimulated HSCs. (A) Protein expressions and quantitative results of IL-1R1, IL-6, Caspase-1, cleaved-Caspase-1 and mature-IL-1*β* in HSC-T6 cells. (B) Effect of PIP on mRNA expressions of *Caspase-1*, *IL1A*, *TNF*, and *IL6* in activated LX-2 cells induced by TGF-*β*. (C) Flow cytometry staining of RAW264.7 cells for iNOS and CD206. (D) Protein expressions and quantitative results of cleaved-Caspase-3. (E) TUNEL staining of AML-12 cells (× 400, scale bar: 50 μm). Each band from Western blotting analysis was normalized against GAPDH (*n* = 3). RT-PCR analysis was normalized against *GAPDH* (*n* = 3). ^###^*P* < 0.001 *vs* normal group; *P < 0.05, ^**^P < 0.01, ^***^P < 0.001 *vs* TGF-*β* or LPS group; ns, no significance.

Inflammation is the main factor in the development of liver fibrosis, and activating HSCs can produce pro-inflammatory mediators leading to a chronic cycle of inflammation, ultimately leading to organ failure (de Carvalho Ribeiro and Szabo, 2022, Hammerich and Tacke, 2023). In comparison to the normal group, TGF-*β* significantly upregulated the protein expressions of IL-1R1, IL-6, Caspase-1 and IL-1*β* in HSC-T6 cells, while PIP inhibited these protein expressions (Fig. 6A). Furthermore, the mRNA expressions of *Caspase-1*, *IL1A*, *TNF* and *IL6* were elevated in response to TGF-*β* stimulation, while PIP decreased these mRNA expressions in LX-2 cells (Fig. 6B). The results suggested that PIP was able to modulate the expressions of inflammatory cytokines in activated HSCs.

The macrophage-mediated inflammatory response plays a vital role in advancing liver fibrosis. Within the liver environment, macrophages have the ability to attract additional inflammatory cells to the injured liver and secrete a range of profibrogenic substances, such as cytokines and chemokines (Jiang et al., 2025). The impact of PIP on macrophage polarization was identified using flow cytometry. LPS significantly promoted the polarization of RAW264.7 cells towards M_1_, while PIP inhibited this effect and reduced the expression of iNOS. PIP resulted in a reduction in the M_1_/M_2_ macrophage ratio, indicating that it efficiently induced a transition of M_1_ to M_2_ macrophages (Fig. 6C). These results indicate that PIP can regulate liver fibrosis by inhibiting macrophage M_1_ polarization. Dying hepatocytes release pro-inflammatory and pro-fibrogenic cytokines that activate HSCs, leading to their activation, extracellular matrix deposition, and ultimately, liver cirrhosis. Hepatocyte apoptosis represents a critical event in hepatic fibrosis progression. PIP significantly reduced the upregulation of cleaved-Caspase-3 protein expression induced by LPS in AML-12 cells (Fig. 6D). In addition, TUNEL analysis exhibited an increase of apoptotic cell numbers in LPS-induced AML-12 cells, while PIP decreased apoptotic cell numbers (Fig. 6E). These results indicated that PIP can inhibit the development of liver fibrosis by alleviating hepatocyte apoptosis.

### 3.6 PIP mediated the JAK2/STAT3 signaling pathway in TGF-β-stimulated HSCs

**Fig. 7:**
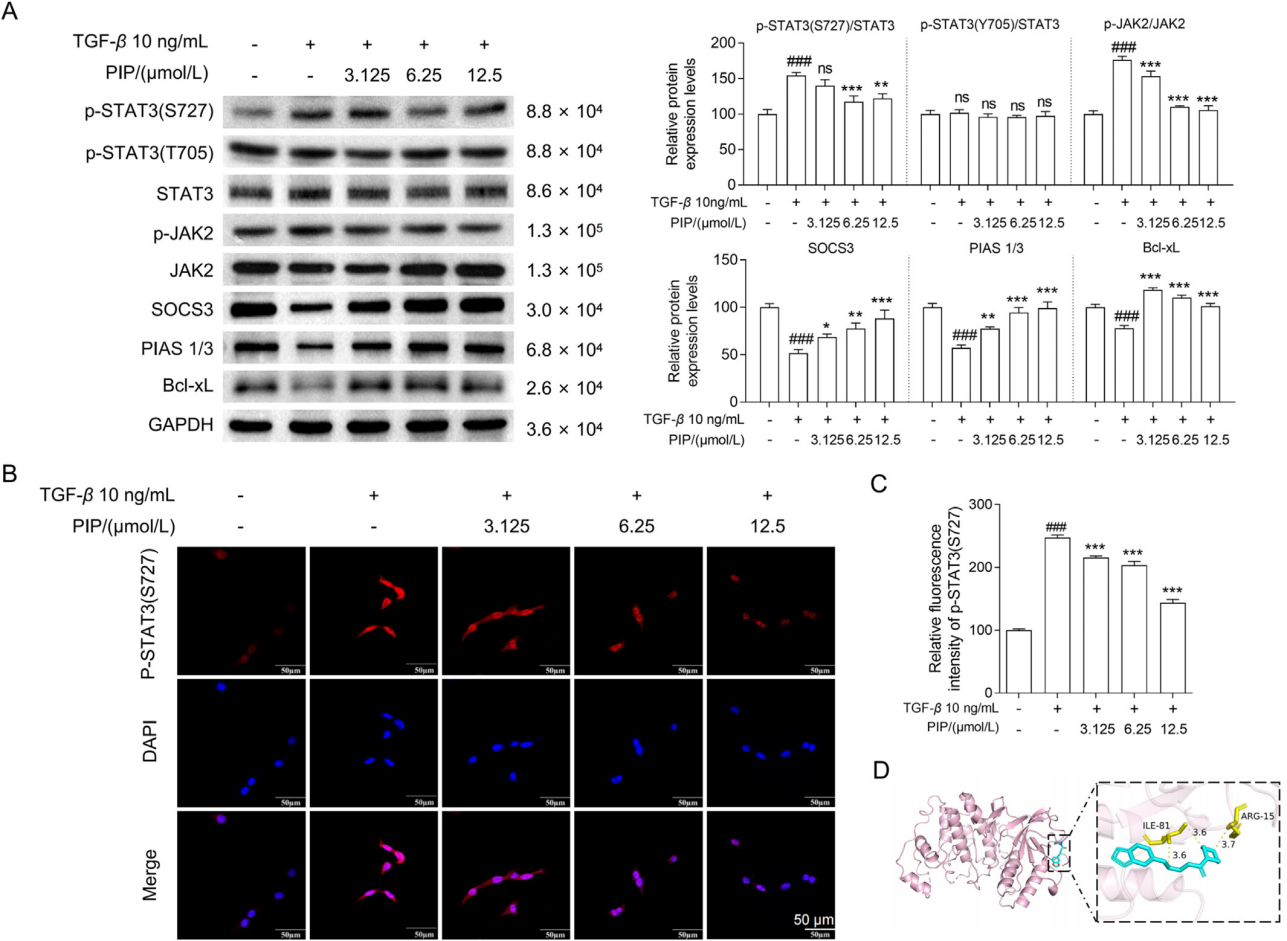
PIP regulates JAK2/STAT3 signaling pathway in activated HSC-T6 cells. (A) Protein expressions and quantitative results of p-STAT3 (Ser727)/STAT3, p-STAT3 (Tyr705)/STAT3, p-JAK2/JAK2, SOCS3, PIAS1/3 and Bcl-xL in HSC-T6 cells. (B) Immunofluorescence staining for p-STAT3 (Ser727) (red, ×600, scale bar: 50 μm), DAPI (blue, ×600, scale bar: 50 μm) and merge presents (×600, scale bar: 50 μm). (C) Relative fluorescence intensity of p-STAT3 (Ser727) was quantified using Image-J. (D) Molecular docking of PIP and STAT3 protein. Each band from Western blotting analysis was normalized against GAPDH (*n* = 3). ^###^*P* < 0.001 *vs* normal group; *P < 0.05, ^**^P < 0.01, ^***^P < 0.001 *vs* TGF-*β* group; ns, no significance.

TGF-*β* significantly upregulated the expressions of p-STAT3 (Ser727) and p-JAK2 in comparison to the normal group, while PIP markedly diminished these protein expressions. TGF-*β* decreased the protein expressions of SOCS3 and PIAS1/3 in comparison to the normal group, while PIP significantly increased the protein expressions of SOCS3 and PIAS1/3. Furthermore, compared with normal group, TGF-*β* significantly reduced the protein expression of Bcl-xL in activated HSC-T6 cells, while PIP increased the protein expression of Bcl-xL. PIP had no significant effect on the protein expressions of p-STAT3 (Tyr705) and total STAT3 (Fig. 7A), which was consistent with the conclusion that had obtained from animal experiments. In comparison to the normal group, immunofluorescence staining of p-STAT3 (Ser727) revealed that the positive expression (in red) of p-STAT3 (Ser727) was notably increased in activated HSC-T6 cells, while the positive expressions were inhibited with PIP (Fig. 7B and C). Molecular docking results showed that the docking energy between PIP and STAT3 is −6.5 kcal/mol (Fig. 7D). The results suggested that PIP could ameliorate hepatic fibrosis by inhibiting STAT3 phosphorylation and mediating the JAK2/STAT3 signaling pathway. The results suggested that PIP could ameliorate hepatic fibrosis by selectively inhibiting STAT3 phosphorylation at Ser727 rather than Tyr705 and following cascade reaction, which was consistent with the conclusion *in vivo*.

### 3.7 PIP may act as STAT3 antagonist to regulate STAT3 (Ser727) phosphorylation via JAK2/STAT3 signaling pathway to ameliorate liver fibrosis

**Fig. 8:**
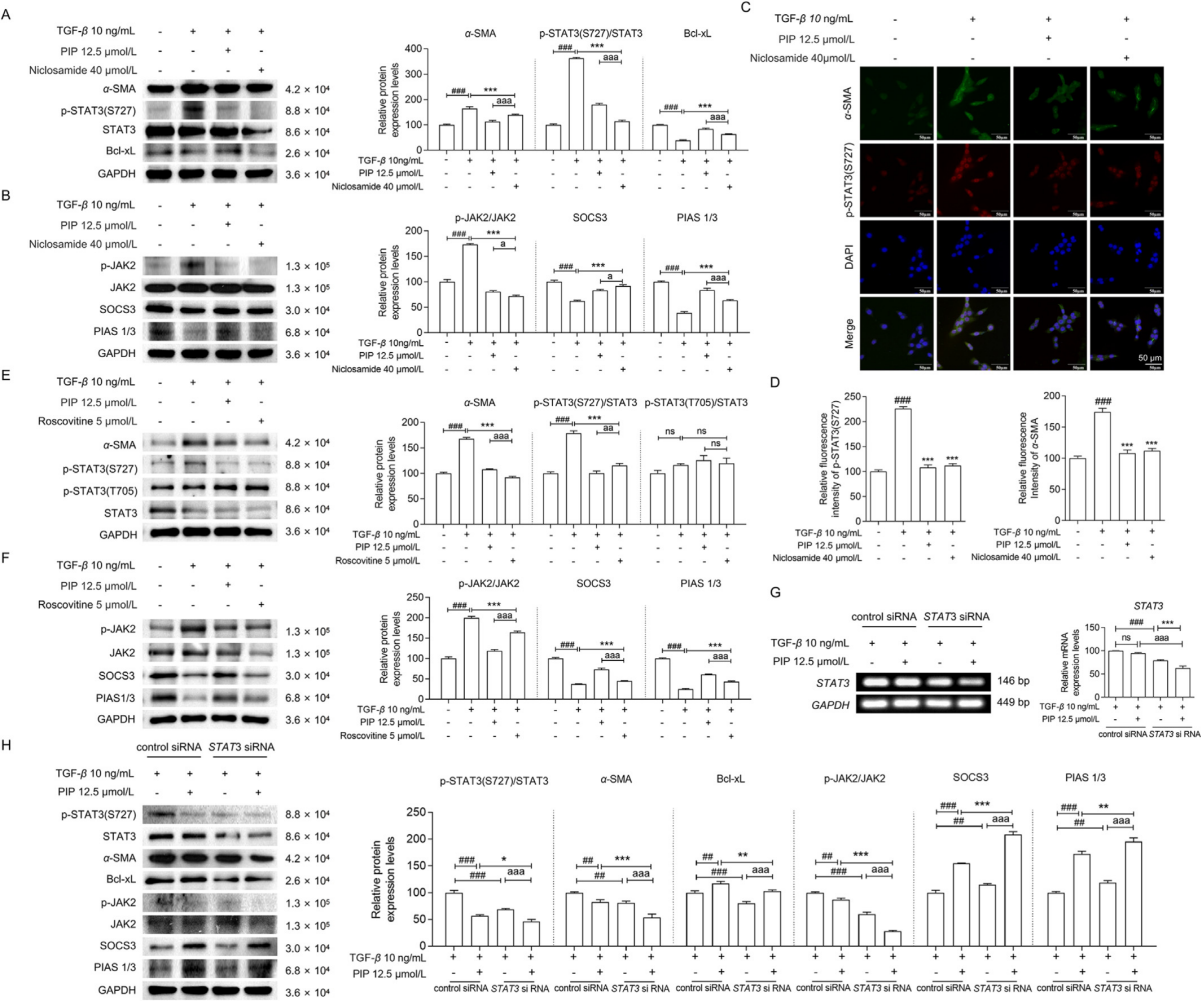
Special targeting JAK2/STAT3 signaling may be a potential therapeutical strategy for PIP against hepatic fibrosis. (A) Protein expressions and quantitative results of *α*-SMA, p-STAT3 (Ser727)/STAT3 and Bcl-xL. (B) Protein expressions and quantitative results of p-JAK2/JAK2, SOCS3 and PIAS1/3. (C) Immunofluorescence staining for *α*-SMA (green, ×600, scale bar: 50 μm), p-STAT3 (Ser727) (red, ×600, scale bar: 50 μm), DAPI (blue, ×600, scale bar: 50 μm) and merge presents (×600, scale bar: 50 μm). (D) Relative fluorescence intensity of *α*-SMA and p-STAT3 (Ser727) was quantified using Image-J. (E) Protein expressions and quantitative results of *α*-SMA, p-STAT3 (Ser727)/STAT3 and p-STAT3 (Tyr705)/STAT3. (F) Protein expressions and quantitative results of p-JAK2/JAK2, SOCS3 and PIAS1/3. (G) Relative mRNA expressions of *STAT3*. (H) Protein expressions and quantitative results of p-STAT3 (Ser727)/STAT3, *α*-SMA, Bcl-xL, p-JAK2/JAK2, SOCS3 and PIAS1/3. Each band from Western blotting analysis was normalized against GAPDH (*n* = 3). RT-PCR analysis was normalized against *GAPDH* (*n* = 3).Significance for Fig. 8A, B: ^###^*P* < 0.001 *vs* normal group; ^***^P < 0.001 *vs* TGF-*β* group; ^a^*P* < 0.05, ^aaa^*P* < 0.001 *vs* PIP group. Significance for Fig. 8D: ^###^*P* < 0.001 *vs* normal group; ^***^P < 0.001 *vs* TGF-*β* group. Significance for Fig. 8E, F: ^###^*P* < 0.001 *vs* normal group; ^***^P < 0.001 *vs* TGF-*β* group; ^a^*P* < 0.05, ^aaa^*P* < 0.001 *vs* PIP group. Significance for Fig. 8G, H: ^##^*P* < 0.01, ^###^*P* < 0.001 *vs* control siRNA-transfected cells treated with TGF-*β*; *P < 0.05, ^**^P < 0.01, ^***^P < 0.001 *vs* control siRNA-transfected cells treated with TGF-*β* + PIP; ^aaa^*P* < 0.001 *vs STAT3* siRNA-transfected cells treated with TGF-*β*, ns, no significance.

Niclosamide acts as a selective inhibitor of STAT3, preventing DNA replication, by blocking the phosphorylation of STAT3, and its translocation into the nuclear (Wu et al., 2020). To further explore the impact of PIP to ameliorate liver fibrosis in regulating the STAT3 signaling pathway, niclosamide was introduced to inhibit STAT3 in activated HSC-T6 cells for the subsequent experiments. Stimulation with TGF-*β* notably enhanced the protein expressions of p-STAT3 (Ser727) and *α*-SMA in comparison to the normal group, while PIP evidently suppressed these protein expressions. TGF-*β* significantly reduced the expression of Bcl-xL, while PIP or niclosamide significantly increased the expression of Bcl-xL compared with TGF-*β* group, but niclosamide downregulated the expression of Bcl-xL compared with PIP (Fig. 8A). In comparison to the normal group, the stimulation of TGF-*β* resulted in an elevation of the expression of p-JAK2 and a reduction in SOCS3 and PIAS1/3 protein expressions. PIP or niclosamide diminished the protein expression of p-JAK2 while upregulating the protein expressions of SOCS3 and PIAS1/3 (Fig. 8B). Double immunofluorescence staining revealed a significant increase in the positive expressions of p-STAT3 (Ser727) (in red) and *α*-SMA (in green) was notably increased due to TGF-*β* stimulation, and PIP presented the similar inhibition on these positive expressions with niclosamide in HSC-T6 cells (Fig. 8C and D). In order to further investigate the specific regulation of Ser 727 phosphorylation by PIP in regulating the STAT3 signaling pathway, roscovitine, a specific inhibitor of Ser727, was introduced in HSC-T6 cells for subsequent experiments. Compared with the normal group, TGF-*β* stimulation significantly increased the protein expressions of *α*-SMA and p-STAT3 (Ser727), while PIP and roscovitine significantly reduced these expressions. PIP and roscovitine had no significant effect on the protein expression of p-STAT3 (Tyr705), and the effect of PIP was similar to roscovitine (Fig. 8E). Under the stimulation of TGF-*β*, the protein expression of p-JAK2 in HSCs was significantly upregulated, while the protein expressions of SOCS3 and PIAS1/3 were significantly downregulated. However, PIP and roscovitine reversed these changes. Compared with the TGF-*β* group, PIP and roscovitine reduced the protein expression of P-JAK2 and increased the protein expressions of SOCS3 and PIAS1/3 (Fig. 8F). These results suggested that PIP may act as a specific p-STAT3 (Ser727) antagonist when it regulated overly extrasellar matrix, p-JAK2 and p-STAT3 (Ser727) via the JAK2/STAT3 pathway in activated HSCs.

To further assess the specific targeting of PIP via JAK2/STAT3 signaling pathway, specific siRNA was used to transfect LX-2 cells, aiming to silence the *STAT3* gene. To keep the homology of cell and siRNA, LX-2 was used to the following experiments, LX-2 is human hepatic stellate cell lines. In activated LX-2, siRNA of *STAT3* successfully established the *STAT3* deficiency in mRNA level, and PIP further suppressed the mRNA expression of *STAT3* (Fig. 8G). *STAT3* deficiency significantly reduced *STAT3* phosphorylation levels at Ser727, with the inhibitory effect being potentiated by subsequent PIP treatment. In activated LX-2, TGF-*β*-induced elevation of *α*-SMA was further weakened with *STAT3*-deficient, and PIP could additionally inhibit *α*-SMA. The expression of Bcl-xL was decreased by *STAT3* deficiency and PIP reversed this effect, increasing the protein expression of Bcl-xL. However, the upregulation effect of PIP on Bcl-xL expression was weakened. *STAT3* deficiency decreased JAK2 phosphorylation and increased SOCS3 and PIAS1/3, while PIP amplified these changes (Fig. 8H). These results demonstrated that PIP has the potential to improve hepatic fibrosis by modulating the JAK2/STAT3 signaling pathway, specifically by targeting *STAT3* phosphorylation at Ser727.

## 4 Discussion

Hepatic fibrosis is associated with the advancement of chronic hepatic disease (Shao et al., 2022). However, there is a dearth of targeted therapeutic strategies for the reversal of liver fibrosis. Growing evidences highlighted the unique advantages of natural products in liver fibrosis treatment, especially active components from traditional Chinese medicine (Luo et al., 2022). As a natural alkaloid derived from *P. nigrum*, PIP has demonstrated robust hepatoprotective properties in liver diseases (Shu et al., 2021, Sharifi et al., 2023). The current study demonstrated that PIP notably improved liver fibrosis by the improving of serum transaminase, histopathological changes, ECM deposition and inflammation by inhibiting STAT3/JAK2 phosphorylation and activating SOCS3 and PIAS1/3 *in vivo* and *in vitro*. In particular, switching STAT3 phosphorylation at Ser727 could represent a potential therapeutic target for PIP against liver fibrosis.

Targeting reverse of activated HSCs is the key step for prevent and treatment for hepatic fibrosis (Ezhilarasan & Najimi, 2024). The present study demonstrated that PIP significantly attenuates HSC activation and exerts hepatoprotective effects in fibrotic liver tissues through suppression of ECM deposition. Therefore, PIP could serve as a promising option for improving hepatic fibrosis by reversing the activation of HSCs.

Chronic inflammation of the liver and its associated regenerative wound-healing response are key driving factors in liver damage and fibrosis (Calvente et al., 2019, Jiang et al., 2024). After hepatocellular injury occurs, liver macrophages become activated, which in turn prompts the synthesis of pro-inflammatory cytokines and chemokines, comprising MCP-1, TNF-*α*, IL-1*β*, and IL-6 (Wang et al., 2020). Underlying stimulating factors, the recruitment of neutrophils can further exacerbate liver damage and inflammatory response (Tangeten, Zouaoui Boudjeltia, Delporte, Van Antwerpen, & Korpak, 2022). The study indicated that administration of PIP significantly decreased the MPO expression and the release of pro-inflammatory cytokines, including Caspase-1, TNF-*α*, IL-1R1, and IL-6, which was beneficial for alleviating inflammation in HSCs activated by TGF-*β* and mice induced by TAA. In addition, Cur and PIP showed significant efficacy in attenuating TAA-induced liver fibrosis, the superior trends observed with PIP suggest it may possess a potent mechanism of action. Thus, improving the hepatic inflammatory response might serve as an additional effective approach for PIP in combating liver fibrosis.

Research has shown that STAT3 can act as a pre-anti-inflammatory signal to regulate liver inflammation and fibrosis, playing a pivotal role in the development of liver disease (Tang et al., 2021). The two signaling pathways, comprising the SOCS family and the PIAS family members, exert a negative regulation on STATs signals (Li et al., 2023, Zhang et al., 2024). Phosphorylated STAT proteins dimerize and translocate to the nucleus where they control the expression of their target genes, such as SOCS3 (Zanders et al., 2022). PIAS1 and PIAS3 exert an inhibitory effect by direct binding to STAT1 and STAT3 and inhibiting their nuclear translocation (Dziemidowicz et al., 2019). PIAS1/3 suppresses STAT3 nuclear function via SUMOylation, both forming negative feedback with STAT3 (Li, Rasul, Sharif, & Hassan, 2024). The Ser-727 phosphorylation of Stat3 affects the transcriptional activity of its downstream targets like SOCS3, Bcl-xL and Cyclin D1 (Mandal et al., 2014). Consequently, targeting JAK2/STAT3 pathway is viewed as a potentially effective strategy for combating liver fibrosis. To further investigate the precise regulatory impact of PIP on JAK2/STAT3 signaling pathway, niclosamide (STAT3 antagonist) and siRNA of STAT3 were further detected in activated HSCs. PIP targeted the regulation of JAK2 and STAT3 to activated SOCS3 and PIAS1/3 and inhibited ECM accumulation, function as STAT3 antagonist. And STAT3 deficiency led to the decrease of JAK2 phosphorylation and *α*-SMA, the increase of SOCS3 and PIAS1/3, and PIP could further amplify these changes. Notably, PIP improved hepatic fibrosis through regulating the JAK2/STAT3 signaling pathway, especially targeting STAT3 phosphorylation at Ser 727 rather than Tyr 705. While phosphorylation at Tyr705 is indispensable for STAT3 dimerization and nuclear translocation, phosphorylation at Ser727 is increasingly recognized as a key regulator of STAT3′s transcriptional potency, mitochondrial localization, and ability to drive pathological processes such as fibrosis and inflammation (Yang et al., 2020, He et al., 2022). PIP specifically inhibits STAT3 phosphorylation at Ser727 while upregulating the key negative regulators SOCS3 and PIAS1/3. SOCS3 upregulation likely suppresses upstream JAK2 activation, thereby forming a potent feedback loop. More notably, the concomitant upregulation of PIAS1/3, which is known to directly bind and suppress STAT3 transcriptional activity, provides a mechanistic basis for the selective inhibition of Ser727 phosphorylation. This dual enhancement of negative regulators targets both the initiation of signaling through SOCS3-mediated regulation of JAK2 and the execution of signaling through PIAS1/3-mediated regulation of STAT3 Ser727 phosphorylation, providing a clear explanation for PIP’s precise suppression of this pathway and its potent anti-fibrotic effects. The results demonstrated that PIP ameliorated hepatic fibrosis by suppressing STAT3 phosphorylation at Ser727 and following JAK2/STAT3 cascade reaction. These findings, demonstrating that PIP attenuated fibrogenesis and inflammation by switching STAT3 phosphorylation at Ser727, position it as a promising candidate for the treatment of hepatic fibrosis. Beyond its efficacy, PIP’s favorable pharmacokinetic profile enhances its translational potential. Prior studies have shown that PIP achieves significant liver exposure after oral administration, with concentrations substantially higher than in plasma (Ganesh Bhat and Chandrasekhara, 1986, Athukuri and Neerati, 2017). The dosing regimen used in this study (15 − 60 mg/kg) is well within the range that achieves these therapeutic exposure levels in preclinical models. Therefore, the combined evidence of robust efficacy and pharmacokinetics profile provided a strong rationale for further development of PIP. On the whole, the regulation effects of PIP on fibrogenesis and inflammation indicated its clinical application for combating liver fibrosis, warranting further investigation in upcoming research.

## 5 Conclusion

In conclusion, the current study demonstrates the protective effect of PIP on the liver and explores the potential mechanism of PIP in combating hepatic fibrosis. PIP apparently attenuate fibrogenesis and inflammation in hepatic fibrosis mice and inhibit the activation of HSCs by switching STAT3 phosphorylation at Ser727 and following cascade reaction, thereby improving the development of liver fibrosis. These results provide new perspectives on the treatment of liver fibrosis and position PIP as a promising candidate for therapy.

## CRediT authorship contribution statement

**Xu Dai:** Methodology, Investigation, Formal analysis, Writing – original draft. **Yunuo Wu:** Methodology, Investigation, Formal analysis. **Lihua Lian:** Resources. **Zhenyu Cui:** Conceptualization, Resources, Funding acquisition. **Jixing Nan:** Conceptualization, Resources, Supervision. **Yanling Wu:** Conceptualization, Resources, Supervision, Funding acquisition, Writing – original draft, Writing – review & editing.

## Declaration of competing interest

The authors declare that they have no known competing financial interests or personal relationships that could have appeared to influence the work reported in this paper.
